# The Campaign against Measles

**Published:** 1920-01-03

**Authors:** 


### THE CAMPAIGN AGAINST MEASLES.

### Effects and Advantages of the New Regulations.

The rescinding of the Measles Regulation of 1915 by the new Order of 1919, is a step which calls for some explanation, and which at first sight seems to be a retrograde movement. However, the circular (No. 35) from the Minister of Health, which accompanies the Order, shows that though compulsory notification is no longer deemed to be advantageous, no slackening of the campaign against the disease is intended. The point where notification failed seems especially to have been, that only the first case in a household had to be notified by the doctor, the remainder being notified by the parent or guardian, and it is the first case which spreads the disease, and which in many cases the doctor does not see till too late to prevent dissemination of the disease. The present order leaves it optional for the local authorities to maintain compulsory notification, but suggests what certainly seem more reasonable means of dealing with this scourge.

### The Future Scheme.

It is suggested that by means of pamphlets and notices a vigorous propaganda should be carried on, the aim of which is to encourage the parents and guardians of children to notify the local sanitary authorities. Also that similar advice should be given at Infant Welfare Centres, and that some form of lectures may be arranged to the same end. Then health visitors, school nurses, and school attendance officers should be told to report all cases occurring under their observation, and that those who are in contact with parents should also take part in the propaganda campaign described above. These methods are to replace the former compulsory notification.

### Treatment.

As to treatment, the health visitor, on finding a case of measles in her district, is to give the child's parents advice as to the care, nursing, feeding, and isolation of the child, and then to report the case to the local sanitary authorities. She should, at the same time, say whether reasonable provision can be made for the child in its home or whether some action on the part of the local authorities is required. She is to continue the supervision of those cases suitable for home treatment until convalescent. Special visitors for measles alone are not considered to be necessary.

### Nursing.

As to nursing it is suggested that the local district nurse be called in, or a nurse from the sanitary authorities' isolation hospital staff. In the case of most district nurses it is a rule that they shall not attend infectious cases, so some special arrangement will need to be made for this, and the recommendation that nurses may attend their ordinary cases and measles cases, provided they take the usual simple precautions, is not likely to be well received even though it is by no means unreasonable.

It is laid down that the health visitor should be instructed always to advise that a doctor should be called in.

As to institution treatment, it is advised that severe cases, as those complicated with broncho-pneumonia, and cases from bad homes, should be treated in hospital. In this case a number of small wards should be used, so that they can be frequently closed and cleaned, and that any cases of broncho-pneumonia should be promptly isolated from the others. The after-care is to be supervised by the health visitor, except those cases which a convalescent home can take, and these, unfortunately, are likely to be few.

### The Health Visitor's Work.

As measles and its complications rank in the first class as causes of infant mortality, no steps likely to combat them should be neglected, and the present suggestions seem to hold out a much greater promise than did notification. The brunt of the work falls on the health visitor, who is likely to be by far the most important factor in spreading the propaganda, who is avowedly the principal agent for notification, and with whom rests the superintendence of the patients' home welfare. Health visitors already have much to contend with, and this extra work will need to be allowed for. Then the provision of special district nurses for infectious cases will not be without difficulty, and will be expensive. But the money saved by the abolition of notification should be sufficient amply to meet this. Also grants not exceeding half the net expenditure are payable by the Ministry of Health towards salaries and expenses of health visitors engaged in maternity and child welfare work (including measles), towards home nursing of measles cases, towards the provision of beds in institutions, and towards convalescent homes. All the above grants are made in respect of children under five years.

### A Critical Age.

It is well to remember that, in spite of the great number of Army cases so recently encountered, measles is essentially a disease of infancy and early childhood. It is true that adults unprotected by a previous attack are equally susceptible, and this fact accounts for the experiences of more than a few of the colonial troops visiting this country, but in London and, in fact, England generally it may be said that the disease is so universal that few children escape it. The mortality is greatest under three years of age and the highest death-rate is reached in the second year of life, but after five years the mortality is enormously diminished. The concentration of our efforts upon the under-five child is therefore fully justified. Indeed nearly 90 per cent. of the total deaths from measles occur in the vital first five years of existence.

